# Mouse Tryptase Gene Expression is Coordinately Regulated by GATA1 and GATA2 in Bone Marrow-Derived Mast Cells

**DOI:** 10.3390/ijms20184603

**Published:** 2019-09-17

**Authors:** Kinuko Ohneda, Shin’ya Ohmori, Masayuki Yamamoto

**Affiliations:** 1 Department of Pharmacy, Faculty of Pharmacy, Takasaki University of Health and Welfare, Takasaki 370-0033, Japan; omori@takasaki-u.ac.jp; 2 Department of Medical Biochemistry, Tohoku University Graduate School of Medicine, Sendai 980-8573, Japan; masiyamamoto@med.tohoku.ac.jp

**Keywords:** mast cell, tryptase, gene transcription

## Abstract

Mast cell tryptases have crucial roles in allergic and inflammatory diseases. The mouse tryptase genes represent a cluster of loci on chromosome 16p3.3. While their functional studies have been extensively performed, transcriptional regulation of tryptase genes is poorly understood. In this study, we examined the molecular basis of the tryptase gene expression in bone marrow-derived mast cells (BMMCs) of C57BL/6 mice and in MEDMC-BRC6 mast cells. The expression of the *Tpsb2* and *Tpsg1* genes, which reside at the 3′-end of the tryptase locus, is significantly decreased by the reduction of the GATA transcription factors GATA1 or GATA2. Chromatin immunoprecipitation assays have shown that the GATA factors bind at multiple regions within the locus, including 1.0 and 72.8 kb upstream of the *Tpsb2* gene, and that GATA1 and GATA2 facilitate each other’s DNA binding activity to these regions. Deletion of the −72.8 kb region by genome editing significantly reduced the *Tpsb2* and *Tpsg1* mRNA levels in MEDMC-BRC6 cells. Furthermore, binding of CTCF and the cohesin subunit Rad21 was found upstream of the −72.8 kb region and was significantly reduced in the absence of GATA1. These results suggest that mouse tryptase gene expression is coordinately regulated by GATA1 and GATA2 in BMMCs.

## 1. Introduction

Mast cell tryptases are expressed abundantly and are the major component in secretory granules. In humans, elevated levels of tryptase in serum are associated with anaphylaxis [1] and systemic mastocytosis [2]. Mice lacking mast cell tryptase mMCP6 show impaired immunoprotective activity against bacteria [3] and parasite [4] infection, suggesting its important role in host defense.

The genes encoding mast cell tryptase form a cluster of loci on chromosome 16p3.3 and 17A3.3 in humans and mice, respectively. Human tryptase loci contain four genes expressed in mast cells: *TPSG1*, *TPSB2*, *TPSAB1* and *TPSD1*. *TPSG1* encodes γ-tryptase, the only membrane-anchored member of the family. In humans, there are three soluble tryptases—α-, β- (βI, βII and βIII) and δ-tryptase—that are transcribed from three genes, *TPSB2*, *TPSAB1* and *TPSD1*. The βII and βIII isoforms are transcribed from the *TPSB2* gene, whereas the α and βI isoforms are transcribed from the *TPSAB1* gene. In mice, the transcripts from the *Tpsg1*, *Tpsb2* and *Tpsab1* genes are mTMT, mMCP6 and mMCP7, respectively. The mTMT is membrane-anchored, whereas mMCP6 and mMCP7 are soluble tryptases.

A strong linkage disequilibrium has been demonstrated between the *TPSAB1* and *TPSB2* genes, and the expression of these genes is polymorphic [5,6]. In mice, no murine counterpart of the human *TPSD1* gene has been found. Although the overall structure and number of tryptase genes have been well conserved in mammals [7], genomic deletions, mutations and copy number abnormalities are frequently found in both mice and humans [5,8,9,10]. For instance, the expression of mMCP7 is dependent on strain background and is disrupted in C57BL/6 mice [8]. Recently, germline duplications and triplications in the *TPSAB1* gene have been identified, and an increased copy number of the *TPSAB1* gene leads to an elevated basal serum tryptase concentration, which is associated with multisystem disorders in humans [10].

However, while genetic and functional studies have been extensively performed, transcriptional regulation of tryptase genes is less well defined [11]. A basic helix–loop–helix transcription factor, microphthalmia-associate transcription factor (MITF), was shown to activate the transcription of the *Tpsb2* [12,13], *Tpsab1* [14] and *Tpsg1* [15] genes. Whereas direct binding of MITF to the proximal promoter region was shown for *Tpsb2* and *Tpsg1* [12,15], the *Tpsab1* activation by MITF was mediated by the activation of c-Jun [14]. Regarding the *Tpsb2* activation, polyomavirus enhancer binding protein 2 (PEBP2) physically interacts with MITF and synergistically activates the *Tpsb2* gene transcription [13]. The MITF mRNA and protein levels were recently shown to be reduced upon copper-mediated phosphorylation of MEK1/2 [16].

In addition to MITF, we previously reported that the GATA transcription factors GATA1 and GATA2 are also involved in the tryptase gene regulation [17,18]. We showed that conditional ablation of GATA2 in bone marrow-derived mast cells (BMMCs) resulted in the reduced expression of a number of mast cell-specific genes, including the mast cell tryptase genes *Tpsb2* and *Tpsg1* [18]. In contrast, GATA1-deficient BMMCs unexpectedly exhibited minor phenotypic changes, although a reduction in the expression of *Tpsb2* and *Tpsg1* was also observed [17]. Furthermore, we found a 500-kb region containing seven GATA sites in the 5′ of the tryptase loci at chromosome 17A3.3. This region, referred to as “region A”, was bound by both GATA1 and GATA2 in ChIP assays [17]. However, the molecular mechanisms underlying the GATA factor-mediated tryptase gene activation are largely unknown. 

In the present study, we investigated how GATA1 and GATA2 regulate tryptase gene expression in BMMCs. Because region A resides at the 5′-end of the locus, we hypothesized that the genes on this locus might be coordinately regulated by the GATA factors.

## 2. Results

### 2.1. The Introduction of siRNA Targeting Either GATA1 or GATA2 into BMMCs Leads to a Significant Reduction in Mast Cell Tryptase Gene Expression 

**Figure 1:**
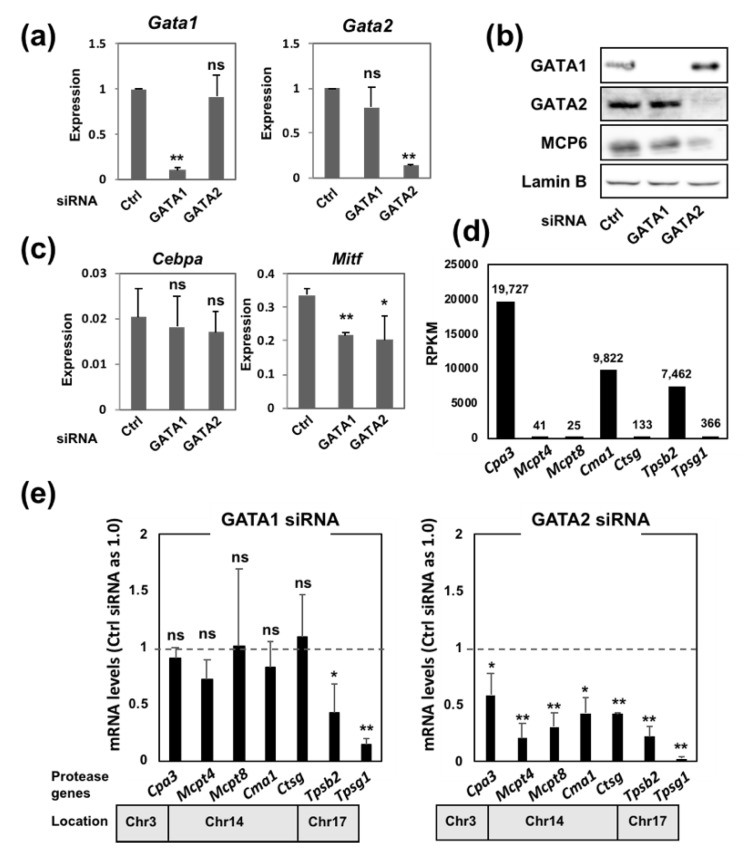
The expression of the mast cell protease gene in the bone marrow-derived mast cells (BMMCs) transfected with either GATA1 or GATA2 siRNA. (**a**) The results of qRT-PCR of GATA1 and GATA2 in the BMMCs transfected with either control, GATA1 or GATA2 siRNA. (**b**) Western blot analyses of GATA1, GATA2, MCP6 and Lamin B (loading control) in the BMMCs transfected with either control, GATA1 or GATA2 siRNA. (**c**) The results of qRT-PCR of C/EBPα and microphthalmia-associate transcription factor (MITF) in the BMMCs transfected with either control, GATA1 or GATA2 siRNA. (**d**) The reads per kilobase million (RPKM) values of mast cell proteases obtained from RNA-seq data of control BMMCs [18]. (**e**) The results of qRT-PCR of mast cell proteases in the BMMCs transfected with the GATA1 or GATA2 siRNAs. The values from the control siRNA-transduced BMMCs were set at 1.0. The bottom bar indicates the localization of protease genes on mouse chromosome. *, *p* < 0.05; **, *p* < 0.01; n.s., not significant. *n* = 3.

To precisely evaluate the contribution of GATA1 and GATA2 to mast cell protease gene expression, siRNA targeting either GATA1 or GATA2 was introduced into BMMCs, and the mRNA levels of mast cell protease genes were assessed by reverse transcription quantitative polymerase chain reaction (qRT-PCR). The introduction of GATA1 and GATA2 siRNAs led to a significant reduction in the corresponding GATA factor expression at both the mRNA and protein levels at 24 h after transfection (Figure 1A,B). In our previous study, the persistent loss of GATA2 led to the dedifferentiation of BMMCs to immature myeloid-like cells with the induction of the myeloid transcription factor C/EBPα [18]. However, at 24 h after siRNA transduction, the C/EBPα mRNA level was not increased by GATA2 ablation (Figure 1C). The MITF mRNA level was moderately but significantly reduced in both GATA1 and GATA2 knockdown cells (Figure 1C). Because the mRNA levels of mast cell proteases at the steady state vary widely, we utilized our previously published RNA sequencing (RNA-seq) data of control BMMCs [18] and checked the reads per kilobase million (RPKM) values of several mast cell proteases (Figure 1D). This revealed that mRNA transcripts from the *Cpa3* gene were the most abundant, followed by those from the *Cma1* and *Tpsb2* genes. 

We then examined the mRNA levels of mast cell protease genes at 24 h after GATA1 or GATA2 siRNA transduction (Figure 1E). Consistent with our previous observation in the conditional GATA2 knockout BMMCs [18], the downregulation of GATA2 resulted in a significant reduction in all mast cell protease genes compared to those transfected with the control siRNA (Figure 1E). In contrast, the downregulation of GATA1 did not affect the expression of *Cpa3* or protease genes located on chromosome 14 (*Mcpt4*, *Mcpt8*, *Cma1* and *Ctsg*). In contrast, consistent with our previous findings for GATA1 knockout BMMCs [17], the expression of mast cell tryptases (*Tpsb2* and *Tpsg1*) was significantly reduced in the GATA1 knockdown cells (Figure 1E). These results support our previous conclusion that GATA2 plays a more important role than GATA1 in the regulation of the mast cell gene expression. GATA1 may have a unique role that cannot be compensated for by GATA2 in the regulation of mast cell tryptase genes.

### 2.2. Overexpression of MITF Failed to Restore the Reduced Tpsb2 Transcript Level in the GATA1/GATA2 Double-Knockdown BMMCs

**Figure 2:**
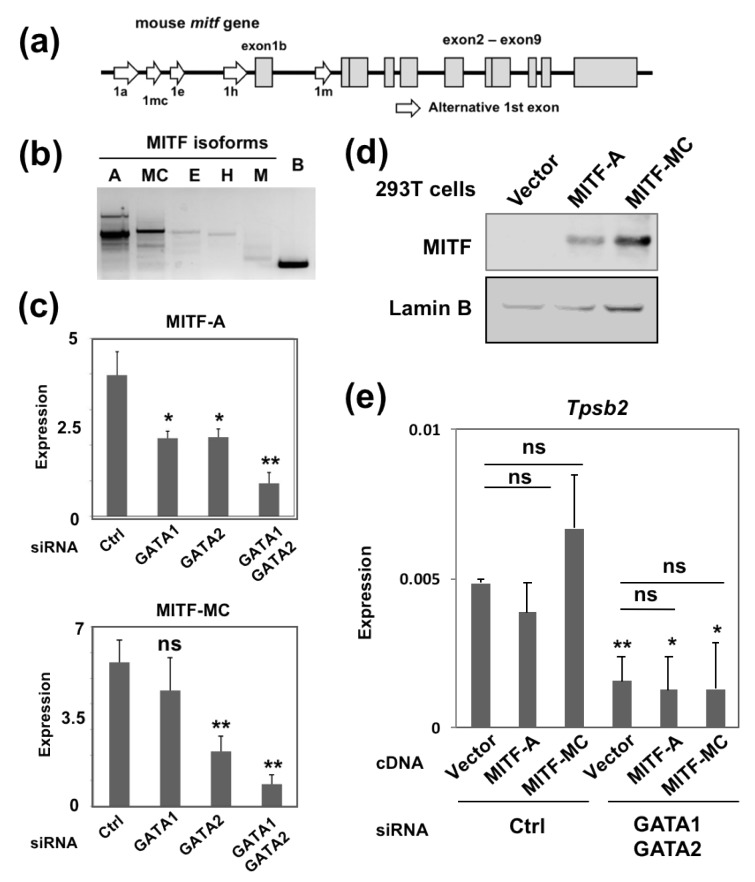
The GATA factor-mediated *Tpsb2* gene repression is independent of MITF. (**a**) A schematic diagram of the mouse MITF gene. Shaded boxes indicate exon 1b and exons 2–8, and open arrows indicate alternative first exons. (**b**) The results of RT-PCR of the MITF isoforms in BMMCs. (**c**) The results of qRT-PCR of MITF-A and MITF-MC isoforms in the BMMCs transfected with the indicated siRNAs. (**d**) Western blot analyses of MITF and Lamin B (loading control) in the 293T cells transfected with either MITF-A or MITF-MC cDNA. (**e**) The results of qRT-PCR of MCP6 in the BMMCs co-transfected with the indicated siRNAs and pIRES2 DsRed-Express2 plasmids. *, *p* < 0.05; **, *p* < 0.01; n.s., not significant. *n* = 3.

Since the MITF mRNA level was reduced in the GATA factor knockdown cells (Figure 1C), we considered that GATA factors might indirectly regulate the *Tpsb2* gene expression through MITF. The mouse *Mitf* gene is transcribed into multiple RNA species by alternative splicing (Figure 2A). These variants differ in their amino termini and are expressed in cell type-specific manners [19]. RT-PCR using a forward primer specific to each alternative first exon and a common reverse primer set at exon 2 revealed that MITF-A and MITF-MC, transcribed from exons 1a and 1mc, respectively, are expressed in BMMCs (Figure 2B). The mRNA levels of both MITF-A and MITF-MC were reduced in GATA1 or GATA2 knockdown BMMCs, although the reduction of MITF-MC mRNA in the GATA1 knockdown cells was not statistically significant (Figure 2C). Simultaneous knockdown of GATA1 and GATA2 in BMMCs (GATA1/GATA2 KD BMMCs) further decreased the MITF-A and MITF-MC mRNA levels, indicating that GATA1 and GATA2 coordinately regulate the *Mitf* gene expression (Figure 2C). To examine whether or not GATA factors regulate the *Tpsb2* gene expression through MITF, MITF-A or MITF-MC cDNA was overexpressed into the GATA1/GATA2 KD BMMCs, and the transcript level of *Tpsb2* gene was examined by qRT-PCR. To this end, the expression plasmids were constructed by using the bicystronic pIRES2 DsRed-Express2 vector, and MITF-A and MITF-MC protein expression was confirmed by Western blot analysis in 293T cells (Figure 2D). These plasmids were co-transfected with the GATA1 and GATA2 siRNAs, and the cells expressing DsRed were sorted for the analysis. This revealed that the overexpression of neither MITF-A nor MITF-MC cDNA affected the *Tpsb2* transcript level in the GATA1/GATA2 KD BMMCs (Figure 2E). These results suggest that GATA1 and GATA2 activate the *Tpsb2* gene expression independent of MITF.

### 2.3. GATA1 and GATA2 Bind to Three Genomic Regions Upstream of the Tpsb2 Gene

**Figure 3:**
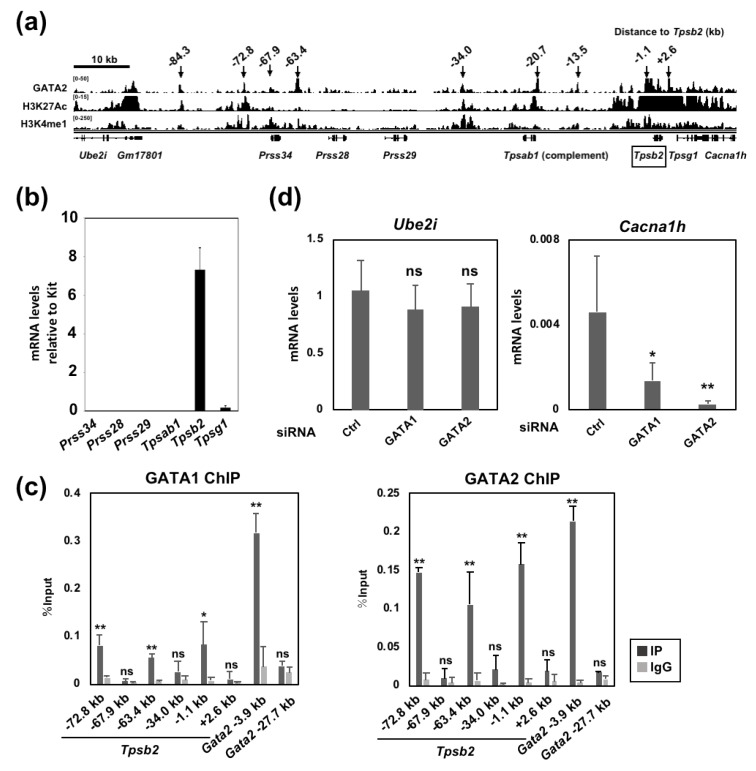
GATA1 and GATA2 bind to multiple regions at the tryptase locus. (**a**) Publicly available ChIP-seq data of the GATA2 binding and the histone modification marks at the tryptase locus in BMMCs [20,21]. The peaks are visualized with the Integrative Genomics Viewer (IGV). (**b**) The results of the qRT-PCR analysis of the genes on mouse chromosome 17A3.3 in BMMCs. The mRNA levels were normalized relative to the level of Kit mRNA. *n* = 3. (**c**) Binding of GATA1 and GATA2 to the tryptase locus was examined by qChIP assays. Chromatin fragments were prepared from wild-type BMMCs. The values of PCR amplicons using immunoprecipitated with an antibody (IP) or the corresponding normal IgG (IgG) relative to those of the input are shown. The regions 3.9 and 27.7 kb upstream of the *Gata2* gene were amplified as positive and negative control regions for GATA2 binding, respectively. In comparison to IgG: *, *p* < 0.05; **, *p* < 0.01; n.s., not significant. *n* = 6. (**d**) The results of qRT-PCR of Ube2i and Cacna1h gene transcript in the BMMCs transfected with the indicated siRNAs. *, *p* < 0.05; **, *p* < 0.01; n.s., not significant. *n* = 3.

To identify cis-acting elements required for the GATA factor-mediated mast cell tryptase gene regulation, we used publicly available ChIP-seq data for GATA2 binding and two histone modifications (H3K27Ac and H3K4me1) that are known to be associated with active enhancers [20,21]. As shown in Figure 3A, several GATA2 binding peaks were observed at the tryptase gene locus on mouse Chr.17A3.3. Some of these peaks were found to overlap with the H3K27Ac and H3K4me1 marks, suggesting that these regions might contribute to the GATA factor-mediated tryptase gene expression. This locus contains six genes (*Prss34*, *Prss28*, *Prss29*, *Tpsab1*, *Tpsb2* and *Tpsg1*) within a region of less than 80 kb. We evaluated the mRNA levels of these genes by qRT-PCR in BMMCs and quantified the relative expression level to that of KIT (Figure 3B). The *Tpsb2* gene was found to be expressed the most abundantly, whereas the *Tpsg1* transcript was detected at a much lower level. Transcripts from the *Prss34*, *Prss28*, *Prss29* and *Tpsab1* genes were undetectable in BMMCs. We named the GATA2 binding peaks of the ChIP-seq data after the distance from the *Tpsb2* gene transcription start site (Figure 3A). Region A, which was bound by GATA1 in our previous report [17], was renamed as the −72.8 kb region in the present study (Figure 3A).

To examine whether or not these GATA2 binding regions were also bound by GATA1, qChIP-PCR was performed using either GATA1 or GATA2 antibody (Figure 3C). The −3.9 and −27.7 kb regions of the *Gata2* gene were amplified as positive and negative controls for GATA factor binding, respectively [22]. We found that the −72.8, −63.4 and −1.1 kb regions were bound by GATA2, and their percent input values were similar to that of the *Gata2* −3.9 kb region. GATA1 binding was observed at similar regions, although the percent input values were smaller than that of the *Gata2* −3.9 kb region. The DNA binding of both GATA1 and GATA2 was weak or undetectable at the −67.9, −34.0 and +2.6 kb regions. Although additional peaks of GATA2 binding were observed at −84.3, −20.7 and −13.5 kb in the ChIP-seq data (Figure 3A), the PCR products were not observed despite the use of two different primer sets. 

The −72.8 and −1.1 kb regions are localized near the 5′ and 3′ ends of the tryptase gene cluster, respectively (Figure 3A). Both regions are bound by the GATA factors and overlap with the ChIP-seq marks for H3K27Ac and H3K4me1. These observations prompted us to examine whether or not the expression of the genes localized outside of the tryptase gene cluster was also influenced by GATA factors. 

Contrary to our hypothesis, the mRNA level of the 5′-neighboring *Ube2i* gene encoding the SUMO-conjugating enzyme UBC9 was not changed in BMMCs treated with either GATA1 or GATA2 siRNA (Figure 3D). In contrast, that of Cacna1h, the 3′ neighboring gene of Tpsg1 encoding a voltage-dependent T-type calcium channel, was significantly decreased in the GATA1 or GATA2 knockdown BMMCs (Figure 3D). These data suggest that the expression of *Cacna1h*, but not *Ube2i*, is regulated by the GATA factors. It is also possible that −72.8 kb region may function as a unidirectional regulatory domain, and the GATA factor binding to this region might affect the expression of downstream (*Tpsb2*, *Tpsg1* and *Cacna1h*), but not upstream (*Ube2i*), genes. Alternatively, although this region is bound by the GATA factors, this region may not have a significant role in gene regulation. 

### 2.4. The GATA2 Binding Activity to the −72.8 kb Region is Reduced by GATA1 Ablation

**Figure 4:**
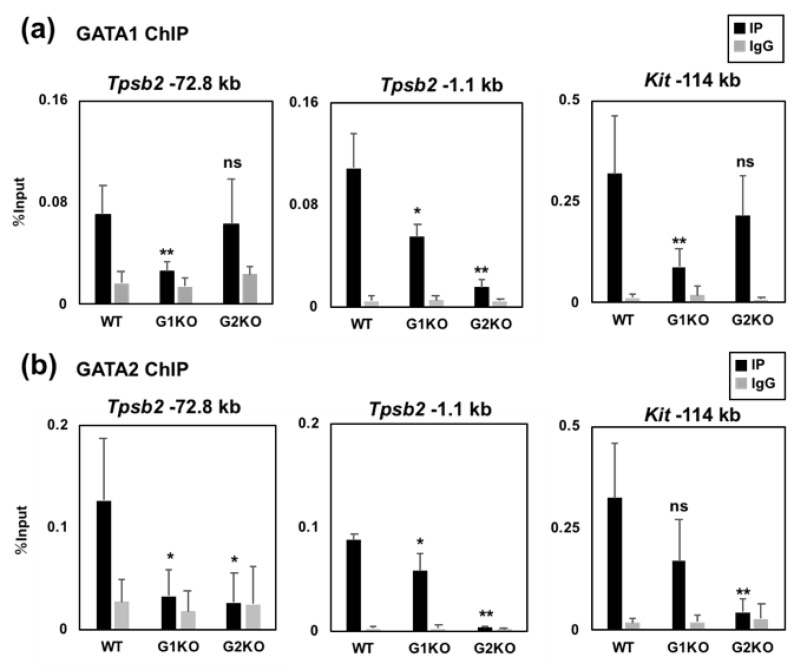
GATA1 and GATA2 affect each other’s binding activity to the −72.8 and −1.1 kb regions. (**a**,**b**) Binding of GATA1 (**a**) and GATA2 (**b**) to the −72.8 and −1.1 kb regions was examined by ChIP-qPCR assays. Chromatin fragments were prepared from either wild-type (WT), G1KO, or G2KO BMMCs. The values of PCR amplicons using immunoprecipitated with an antibody (IP) or the corresponding normal IgG (IgG) relative to those of the input are shown. The results were obtained from four independent assays. The region 114 kb upstream of the *Kit* gene was amplified as a positive control region for GATA1 and GATA2 binding. *, *p* < 0.05; **, *p* < 0.01; n.s., not significant. *n* = 4.

To further examine the DNA binding specificity of GATA1 and GATA2 to the −72.8 and −1.1 kb regions, we utilized BMMCs prepared from conditional knockout mice of GATA1 (G1KO) and GATA2 (G2KO) and examined whether or not GATA1 and GATA2 affect each other’s DNA binding activity to these regions. These mice express *Rosa26CreER^T2^* gene and their gene recombination is induced by the 4-OHT treatment in BMMCs [17,18]. We previously observed that the Cre-loxP-mediated gene recombination of the GATA1 flox allele takes time, and this is possibly due to the long distance between the two loxP sites (approximately 6.8 kb) [17]. In contrast, the recombination of the GATA2 flox allele, which only excises exon 5 encoding the DNA binding domain, occurs rapidly [18]. The *Kit* −114 kb region was examined as a positive locus for GATA factor binding [17]. The GATA factor binding to the −72.8 and −1.1 kb upstream regions was significantly reduced in the corresponding gene knockout BMMCs, while the residual GATA1 protein might affect the DNA binding activity (Figure 4A,B). Interestingly, the GATA2 binding to the −72.8 and −1.1 kb regions were significantly reduced in the G1KO BMMCs (Figure 4B). In particular, the reduction of GATA2 binding to the −72.8 kb region in the G1KO BMMCs was comparable to that of the G2KO BMMCs. These results indicate that GATA1 is required for the maximal binding activity of GATA2 to the −72.8 and −1.1 kb regions. In contrast to GATA1, GATA2 ablation did not affect the GATA1 binding to the −72.8 kb region (Figure 4A). In contrast, the GATA1 binding activity to the −1.1 kb region was reduced in the G2KO BMMCs, and the percent input value was even less than that in the G1KO BMMCs (Figure 4A). Such cross regulation of the DNA binding activity was not observed at the Kit −114 kb region. (Figure 4A,B). 

Taken together, these results indicate that GATA1 and GATA2 affect each other’s binding activity to the −72.8 and −1.1 kb regions. In addition, GATA1 might have a critical role in GATA2 binding to the distal −72.8 kb region, whereas GATA2 might be required for GATA1 binding to the proximal −1.1 kb region.

### 2.5. The −72.8 kb Upstream Region of the Tpsb2 Gene is Indispensable for the Expression of the Tpsb2 and Tpsg1 Genes in MEDMC-BRC6 Murine Mast Cells

**Figure 5:**
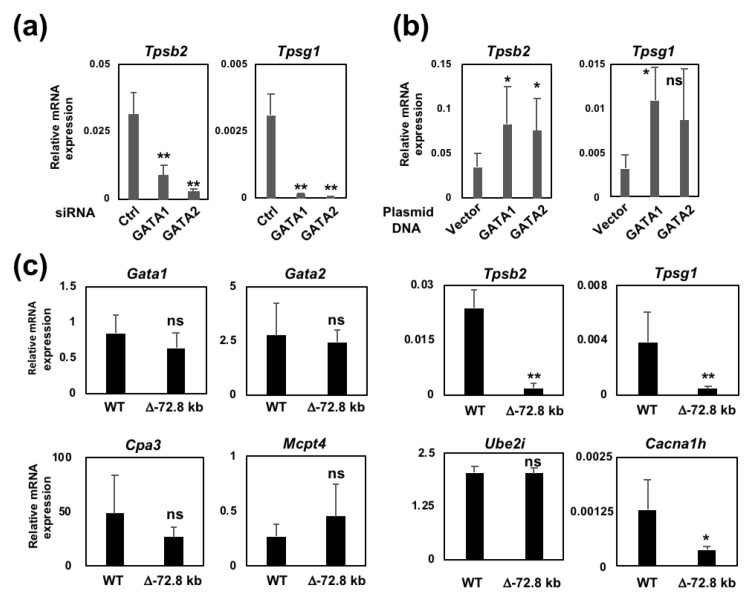
The deletion of the −72.8 kb region reduced the *Tpsb2* and *Tpsg1* gene expression in BRC6 mast cells. (**a**,**b**) Results of qRT-PCR of the *Tpsb2* and *Tpsg1* gene transcript in BRC6 cells transfected with either control, GATA1 or GATA2 siRNA (**a**) or pEF plasmid DNAs (**b**). In the plasmid transfection, pmaxGFP was co-transfected with pEF plasmid DNAs, and green fluorescent protein (GFP)-positive cells were sorted at 24 h after transfection. (**c**) The results of qRT-PCR of mRNAs transcribed from the indicated genes in genome-edited BRC6 cells. The samples were prepared from undeleted (WT) and homozygous (Δ−72.8 kb) clones. *n* = 6 for each group. *, *p* < 0.05; **, *p* < 0.01; n.s., not significant. *n* = 5.

To determine whether or not the −72.8 kb region contributes to the *Tpsb2* and *Tpsg1* gene expression, this region was deleted using CRISPR/Cas9-mediated genome editing in MEDMC-BRC6 (BRC6) murine mast cells [23]. qRT-PCR analyses showed that transcripts from *Tpsb2* and *Tpsg1* were significantly decreased in the GATA1 or GATA2 knockdown cells (Figure 5A) and increased in the cells transfected with an expression plasmid encoding either GATA1 or GATA2 cDNA (Figure 5B). These results suggest that the expression of these genes was dependent on the GATA factors in BRC6 cells, as observed in BMMCs.

Using CRISPR/Cas9-mediated genome editing, the −72.8 kb region was deleted and replaced with a homologously recombined *PGK-gb2-neo* gene cassette. As a result, we obtained 6 homozygous clones (Δ−72.8 kb) that were used for the qRT-PCR analysis (Figure 5C). The G418 resistant clones in the absence of the −72.8 kb deletion were examined as wild-type controls. The GATA1 and GATA2 mRNA levels were unaffected by the −72.8 kb deletion. Notably, both the *Tpsb2* and *Tpsg1* transcript levels were significantly reduced in the Δ−72.8 kb clones. In contrast, the −72.8 kb deletion did not markedly affect either the *Cpa3* or *Mcpt4* transcript levels, denying any non-specific effects on the mast cell phenotype in the Δ−72.8 kb clones. Interestingly, the transcript level of *Cacna1h*, but not *Ube2i*, was significantly lower in the Δ−72.8 kb clones than in the wild-type clones, as was observed in the GATA factor knockdown BMMCs (Figure 5C). Taken together, these data suggest that the −72.8 kb region is required for the expression of far downstream *Tpsb2*, *Tpsg1* and *Cacna1h* genes, whereas it is dispensable for the upstream *Ube2i* gene expression.

### 2.6. GATA1 Regulates the CTCF and Rad21 Binding Activity to the Tryptase Gene Locus 

**Figure 6:**
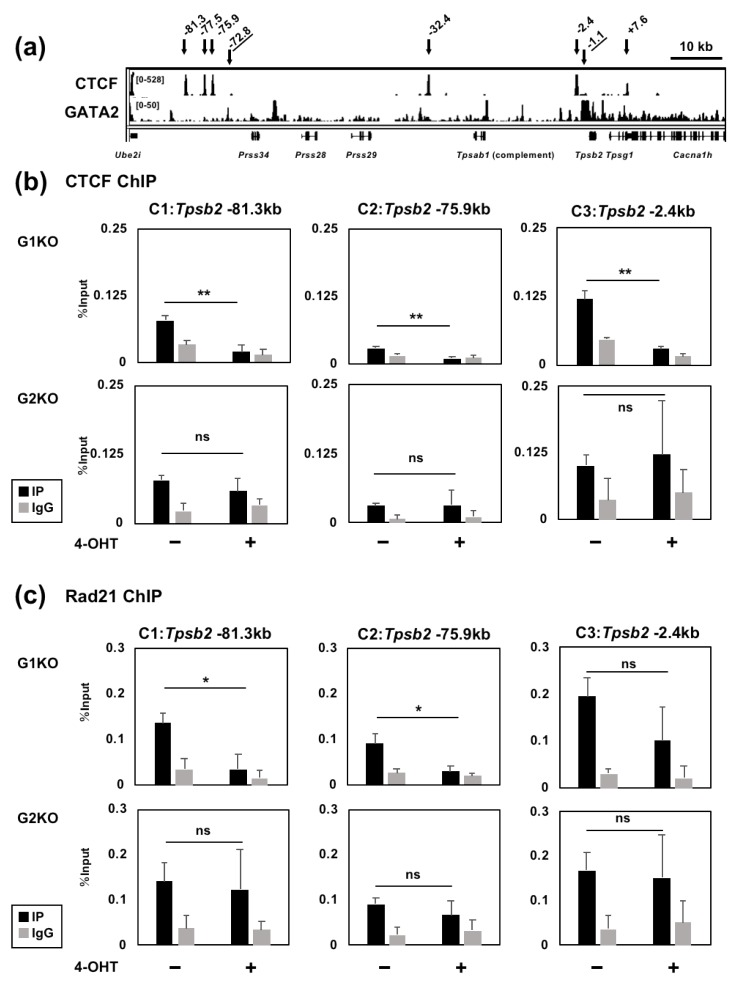
Conditional ablation of GATA1 in BMMCs resulted in a significant reduction in the CTCF binding to the −81.3 and −75.9 kb regions. (**a**) Publicly available ChIP-seq data of the CTCF and GATA2 binding at the tryptase locus in BMMCs. The peaks are visualized with the IGV. (**b**,**c**) Genomic binding of CTCF (**b**) and Rad21 (**c**) to 81.3, 75.9 and 2.4 kb upstream of the *Tpsb2* gene was examined by the ChIP-qPCR assays. Chromatin fragments were prepared from G1KO and G2KO BMMCs cultured either in the presence or absence of 4-OHT. The values of PCR amplicons using immunoprecipitated with an antibody (IP) or the corresponding normal IgG (IgG) relative to those of the input are shown. The results were obtained from four independent assays. *, *p* < 0.05; **, *p <* 0.01; n.s., not significant. *n* = 4.

Our data showed that the −72.8 kb region functions as a unidirectional regulatory region for the *Tpsb2* and *Tpsg1* genes, and the GATA2 binding activity to this region is regulated, at least in part, by GATA1. Based on these findings, we surmised that there might be boundary regions that separate the −72.8 kb region and the upstream *Ube2i* gene. Because CTCF is often found in the chromatin domain boundaries [24], we utilized a published ChIP-seq dataset for CTCF binding in BMMCs [20] to visualize the CTCF binding peaks at the tryptase gene locus (Figure 6A). We found three CTCF binding peaks between the −72.8 kb region and the *Ube2i* gene. Additional CTCF binding peaks were observed at −32.4, −2.4 and +7.6 kb upstream of the *Tpsb2* gene. 

CTCF often shares DNA binding sites with cohesion, and the CTCF/cohesin complex plays central roles for creating the chromatin boundaries and the loop formation [25]. Our ChIP analysis revealed that the −81.3, −75.9 and −2.4 kb regions (Figure 6A) are bound by both CTCF and the cohesion subunit Rad21 in BMMCs (Figure 6B,C). Surprisingly, the CTCF binding activities at the −81.3, −75.9 and −2.4 kb regions were significantly decreased in the GATA1, but not GATA2, knockout BMMCs (Figure 6B). The Rad21 binding activity to the −81.3 and −75.9 regions was also reduced by GATA1, but not GATA2, ablation, although the reduction at the −2.4 kb region was unaffected in both mice. In summary, these results suggest that GATA1, but not GATA2, can facilitate the CTCF/Rad21 binding to the 5′-boundary regions of the tryptase locus.

## 3. Discussion

In the present study, we investigated the regulation of mouse tryptase gene expression by GATA factors. Our data suggest that the genes encoding mast cell tryptase on mouse chromosome 17A3.3 are coordinately regulated by GATA factors. Within the mouse tryptase gene locus spanning approximately 75 kb, only two genes (*Tpsb2* and *Tpsg1*) were found to be expressed in the BMMCs of C57BL/6 mice. These genes reside next to each other in the 3′-end of the locus. To our surprise, deletion of the −72.8 kb region that resides in the 5′-end of the locus severely affected the expression of *Tpsb2* and *Tpsg1* genes. The −72.8 kb region contains seven classical GATA recognition sequences, (A/T)GATAA, and two of them match the chromatin occupancy sequence for GATA1, (C/G)(A/T)GATAA(G/A/C)(G/A/C), that was reported in previous ChIP-seq studies [26,27,28]. Of note, deletion of this region affected the expression of the 3′-neighboring *Cacna1h* gene but not the 5′-neighboring *Ube2i* gene. Similar results were also obtained in BMMCs treated with either GATA1 or GATA2 siRNA. The expression of the *Ube2i* gene encoding the SUMO-conjugating enzyme Ubc9 is not mast cell-specific, and its transcript is detected in a variety of tissues [29]. Taken together, these results suggest a possibility that the gene regulatory function of the −72.8 kb region is dependent on the GATA factors and is unidirectional. Further studies that evaluate the expression of other neighboring genes are needed for understanding the function of the −72.8 kb region.

Recently, a chromosome conformation capture (3C) assay and related Hi-C technique reveal that the three-dimensional structure of chromosomes is dynamically regulated, depending on the cell type, stage of development and extracellular condition [30,31]. Eukaryotic chromatin is organized into compartments composed of topologically associating domains (TADs) that are further divided into smaller units termed subTADs [32,33,34,35]. TADs and subTADs are critical chromosome structural domains in the regulation of the long-range gene expression. CTCF and cohesion have been shown to play crucial roles in the formation of TADs and subTADs, and their binding sites are often found at the boundary region of TADs [24]. In the mouse tryptase locus, three binding peaks of CTCF residing between the *Ube2i* gene and the −72.8 kb region were found by the ChIP-seq data, and two of them were bound by CTCF and Rad21 in our ChIP analyses. Considering the unidirectional effect of the −72.8 kb region, we speculate that these regions might function as a barrier to prevent the GATA factor-dependent, mast cell-specific gene activation of the *Ube2i* gene. The 3C and Hi-C analyses in combination with deletion of the CTCF or GATA factor binding regions may help clarify the 3D chromosome architecture of the tryptase locus. In addition, whether or not the −72.8 kb region resides in close proximity to the *Tpsb2* and *Tpsg1* promoters due to chromatin looping should be clarified. 

Our data indicate that GATA1 plays specific roles that cannot be compensated for by GATA2 in the regulation of mast cell tryptase genes. The ChIP analyses showed that GATA1 ablation significantly reduced the GATA2 binding to the distal −72.8 kb region. Given that deletion of the −72.8 kb region affects the expression of *Tpsb2* and *Tpsg1*, GATA1 might be required for the GATA2-mediated activation of the −72.8 kb region. Notably, ablation of GATA1, but not GATA2, affected the CTCF and Rad21 binding to the −81.3 and −75.9 kb regions. Thus, it is possible that GATA1 facilitates the recruitment of CTCF and cohesin to the 5′-region of the tryptase locus and thereby promotes the formation of active chromatin structure. Interestingly, GATA2 ablation attenuated the GATA1 binding to the proximal −1.1 kb region, whereas the binding to the −72.8 kb was not affected. Thus, GATA2 might have a dominant role in activating the *Tpsb2* and *Tpsg1* promoters. 

In erythroid and megakaryocytic cells, the genome-wide DNA binding of GATA1 and GATA2 has been comprehensively studied by ChIP-seq analyses [26,27,28,36,37]. These studies showed that numerous cell type-specific genes were commonly regulated by both factors. Although these studies defined several unique target genes of either GATA1 or GATA2, the difference between the overlapping and specific target gene regulation has not been clearly defined. GATA1 and its cofactor FOG-1 were shown to be required for looping the β-globin locus control region to the active β-globin promoter [38]. Subsequently, several factors that interact with GATA1, such as Ldb1 [39], BRG1 [40] and SCL/TAL1 [41], have shown to play crucial roles for the chromatin looping formation. These findings suggest that GATA1 might interact with a specific partner to promote the formation of active chromatin structure in mast cells. Further investigations will be needed in order to define the functional differences between GATA1 and GATA2 in mast cell-specific gene regulation.

An earlier study showed that the expression of tryptase genes in vivo is restricted to a particular cell type or tissue, and each individual gene has a different expression profile [9]. These data seem to be inconsistent with the present data. In this study, we used BMMCs and the mast cell line BRC6, which are not fully differentiated compared to highly differentiated peripheral tissue mast cells. The coordinated gene expression regulation of the entire tryptase locus, that has been shown in this study, might be restricted to immature mast cells. Alternatively, only when the structure of the entire locus is activated can a local gene regulatory system of an individual gene be formed during differentiation. BMMCs can be differentiated to connective tissue-type mast cells by culturing with Swiss 3T3 fibroblasts [42]. This system can be used to determine whether or not the coordinated gene regulation of the tryptase locus is differentiation stage-dependent.

## 4. Materials and Methods 

### 4.1. Mice

Conditional knockout mice of *Gata1* (*Gata1*^flox/y^) and *Gata2* (*Gata2*^flox/flox^) were kindly provided by S. Philipsen (Erasmus MC, Rotterdam, the Netherlands) and S. A. Camper (University of Michigan, Ann Arbor, MI, USA), respectively [43,44]. The knockin mice expressing a 4-hydroxy tamoxifen (4-OHT)-inducible Cre recombinase gene under the control of the Rosa26 promoter (Rosa26CreER^T2^) were kindly provided by Anton Berns, The Netherlands Cancer Institute. The *Gata1* and *Gata2* knockout phenotype was examined in BMMCs prepared from hemizygous (*Gata1*^−/y^) and homozygous (*Gata2*^−/−^) male mice expressing CreER^T2^. The study was approved by the Animal Experiment Committee of the Takasaki University of Health and Welfare (Kendai1526, 31 Mar 2015, Kendai1601, 1 Apr 2016, Kendai1730, 15 Mar 2017, Kendai1816, 1 Apr 2018 and Kendai1919, 1 Apr 2019). The animal experiments were carried out in accordance with The Guidelines on Animal Experiments in Takasaki University of Health and Welfare, Japanese Government Animal Protection and Management Law (No.105) and Japanese Government Notification on Feeding and Safekeeping of Animals (No.88). The mice were maintained in an animal facility of Takasaki University of Health and Welfare in accordance with institutional guidelines. 

### 4.2. Cell Culture

Bone marrow mononuclear cells isolated from the femurs and tibiae were cultured for two weeks in Roswell Park Memorial Institute (RPMI) 1640 medium supplemented with 10% FBS and penicillin-streptomycin in the presence of 10 ng/mL recombinant murine IL-3 (PeproTech, Rocky Hill, NJ, USA) and subsequently cultured for two weeks with IL-3 and 10 ng/mL recombinant murine stem cell factor (Peprotech). The 293T cells (Thermo Fisher Scientific) were cultured in Dulbecco modified Eagle’s medium (DMEM) supplemented with 10% fetal bovine serum (FBS) and penicillin-streptomycin. MEDMC-BRC6 cells [23] were purchased from RIKEN BRC (Tsukuba, Japan) and cultured in Iscove’s modified Dulbecco’s medium (IMDM; Invitrogen, Thermo Fisher Scientific, Waltham, MA, USA) containing the following: 15% FBS, ITS liquid media supplement (Sigma-Aldrich, St. Louis, MO, USA), 50 mg/mL ascorbic acid (Sigma-Aldrich), 0.45 mM α-monothioglycerol (Sigma-Aldrich), 3 ng/mL IL-3, 30 ng/mL stem cell factor (SCF), 1% penicillin-streptomycin solution and 2 mM l-glutamine (Nacalai Tesque, Kyoto, Japan). 

### 4.3. Transfection of Small Interfering RNA (siRNA) or Plasmid DNA

The siRNA duplexes for mouse GATA1 and GATA2 were purchased from Invitrogen. The control siRNA was purchased from Sigma. In the knockdown experiments, BMMCs (2.0 × 106 cells) were transfected with 200 pmol of siRNA by electroporation using an Amaxa Nucleofector (Lonza, Basel, Switzerland). Cells were harvested 24 h after transfection and used for the analyses. In the co-expression experiments shown in Figure 2, we generated MITF-A and MITF-MC cDNAs by PCR and cloned them into a bicistronic expression plasmid pIRES2 DsRed-Express2 Vector (Takara Bio USA, Mountain View, CA, USA). BMMCs (2.0 × 10^6^ cells) were transfected with 200 pmol of siRNA and 2.5 µg of plasmid DNA by electroporation, and the cells expressing DsRed were sorted using a FACSJazz cell sorter (BD Biosciences, Franklin Lakes, NJ, USA) at 24 h after transfection. In the plasmid transfection experiments shown in Figure 5B, we used the pEF-BOS [45] expression plasmids GATA1 and GATA2 [46], and pmaxGFP vector (Lonza). pEF plasmids (4 µg; GATA1, GATA2, or empty vector) and 1 µg of pmaxGFP were co-transfected into BRC6 cells by electroporation. At 24 h after transfection, GFP-positive cells were sorted using the cell sorter and used for the analyses.

### 4.4. Quantitative RT-PCR (qRT-PCR)

Total RNA was isolated from cells using a NucleoSpin RNA II (Macherey-Nagel, Düren, Germany). The reverse transcription reactions were performed using a ReverTra Ace qPCR RT Kit (Toyobo, Osaka, Japan) according to the manufacturer’s instructions. Quantitative RT-PCR (qRT-PCR) was performed using the Go Taq qPCR Master Mix (Promega, Madison, WI, USA) and an Mx3000P real-time PCR system (Agilent, Santa Clara, CA, USA), as described previously [47]. The results were normalized to the transcript from the polymerase (RNA) II (DNA directed) polypeptide A (*Polr2a*) gene. The primer sequences used for PCR are shown in Table A1. 

### 4.5. Western Blotting

The whole lysates were prepared as previously described (ref). The samples were resolved by 10% sodium dodecyl sulfate polyacrylamide gel electrophoresis, and the Western blot analyses were performed as described previously [47] using anti-GATA1 (N6; Santa Cruz, Dallas, TX, USA), anti-GATA2 (H-116; Santa Cruz), anti-MCP6 (sc-32474; Santa Cruz), anti-MITF (H-50; Santa Cruz), and anti-lamin B (M-20; Santa Cruz) antibodies. 

### 4.6. Chromatin Immunoprecipitation (ChIP) 

A ChIP assay was performed as in a prior report [47] using anti-GATA-1 (N6; Santa Cruz), anti-GATA2 (B9922A; Perseus Proteomics, Tokyo, Japan), anti-CTCF (A300-543A; Bethyl, Montgomery, TX, USA) and anti-Rad21 (Ab992; Abcam, Cambridge, UK) antibodies and normal Rabbit IgG (2729S; Cell Signaling Technology, Danvers, MA, USA) and anti-Rat IgG (sc-2026; Santa Cruz). As described previously, the GATA1 ChIP assay was done using an anti-rat IgG rabbit antibody (Jackson ImmunoResearch, West Grove, PA, USA) as a secondary antibody to precipitate the immune complex [47]. The DNA purified from ChIP samples were amplified was analyzed using an Mx3000P real-time PCR system (Agilent) with the GoTaq qPCR Master Mix (Promega). The primer sequences used are shown in Table A2. 

### 4.7. CRISPR/Cas9 Genome Editing 

To delete the −72.8 kb region using the CRISPR/Cas9 system, we used two Cas9 expression plasmids: pSpCas9(BB)-2A-GFP (PX458) and pSpCas9(BB)-2A-Puro (PX459) V2.0. These plasmids were a gift from Feng Zhang (Addgene plasmid #48139 and #48138) [48]. Single-guide RNAs (sgRNAs) targeting the 5′ and 3′ end of the −72.8 kb region were designed using CRISPRdirect (https://crispr.dbcls.jp). The sgRNA target sequences were synthesized as DNA oligonucleotides, and a pair of annealed oligonucleotides was inserted into either PX458 or PX459 using the golden gate method. To prepare a targeting vector for homologous recombination, the 5′ and 3′ homology arms were amplified by PCR. The resulting 5′ (1.1 kb) and 3′ (1 kb) arms and the PGK-gb2-neo template (Gene Bridges, Heidelberg, Germany) were cloned into a pBluescript KS vector (Agilent Technologies). BRC6 cells were co-transfected with 2 µg each of PX458 and PX459 vectors harboring the 5′ and 3′ gRNAs, respectively, and 1 µg of the targeting plasmid by electroporation using NEPA21 (Nepa Gene, Chiba, Japan). At 24 h after electroporation, the cells were cultured with 1.0 µg/mL of puromycin for another 24 h, and subsequently, the GFP-positive cells were sorted using a FACSJazz cell sorter into 96-well plates. At 24 h after sorting, G418 was added at a concentration of 1.5 mg/mL, and the cells were cultured for 10 days. The genomic deletion was examined by PCR using genomic DNA isolated with PBND buffer (10 mM Tris-HCl, pH 8.3, 50 mM KCl, 2.5 mM MgCl2, 0.1 mg/mL Gelatin, 0.45% NP-40, 0.45% Tween20 and 20 mg/mL Proteinase K). For homozygous deletion mutants, deletion of the −72.8 kb region was further confirmed by DNA sequencing. The sgRNA target sequences and PCR primers used for the arms are shown in Table A3. 

### 4.8. ChIP-seq Data Processing 

The previously described ChIP-seq data sets (GSE48086 and GSE97253) [20,21] were aligned to the mouse genome (mm9 assembly). The data (GATA2, GSM1167578; H3K27Ac, GSM2564722; H3K4me1, GSM2564728; CTCF, GSM1167574) were visualized using the Integrative Genomics Viewer (IGV).

### 4.9. Statistical Analyses

Comparisons between two groups were made using Student’s *t*-test. Data are presented as the means ± standard deviation. For all of the analyses, statistical significance was defined as a *p*-value < 0.05.

## Abbreviations

BMMCs	Bone marrow-derived mast cells
ChIP	Chromatin immunoprecipitation
MITF	Microphthalmia-associate transcription factor
RT	Reverse transcription
qPCR	Quantitative polymerase chain reaction
RNA-seq	RNA sequencing
RPKM	reads per kilobase million
ChIP-seq	chromatin immunoprecipitation sequencing
3C	chromosome conformation capture
TAD	topologically associating domains
4-OHT	4-hydroxy tamoxifen
RPMI	Roswell Park Memorial Institute
IMDM	Iscove’s modified Dulbecco’s medium
FBS	fetal bovine serum
Polr2a	polymerase (RNA) II (DNA directed) polypeptide A
GFP	green fluorescent protein
sgRNA	single-guide RNA
IGV	Integrative Genomics Viewer

## Appendix A

**Table A1:** Primer sequences for qRT-PCR analyses.

Primers for qRT-PCR	Sequences (5′ to 3′)	
	Forward	Reverse
*Gata1*	CAGAACCGGCCTCTCATCC	TAGTGCATTGGGTGCCTGC
*Gata2*	GCACCTGTTGTGCAAATTGT	GCCCCTTTCTTGCTCTTCTT
*Cebpa*	AAAGCCAAGAAGTCGGTGGAC	CTTTATCTCGGCTCTTGCGC
*Mitf*	GCTGGAGATGCAGGCTAGAG	TGATGATCCGATTCACCAGA
*Cpa3*	GCTACACATTCAAACTGCCTCCT	GAGAGAGCATCCGTGGCAA
*Mcpt4*	CATGCTTTGTTGAACCCAAGG	GAAGTGAAAAGCCTGACCTGC
*Mcpt8*	GTGGGAAATCCCAGTG	GACAACCATACCCCAG
*Cma1*	CCTGGGTTCCAGCACCAA	GGCGGGAGTGTGGTATGC
*Ctsg*	GAGTCCAGAAGGGGCTG	GATGGCTCTGAGACAT
*Tpsb2*	CGACATTGATAATGACGAGCCTC	ACAGGCTGTTTTCCACAATGG
*Tpsg1*	GGTCACACTGTCTCCCCACT	GCATCCCAGGGTAGAAGTCA
*Mitf ex1a*	AAGTCGGGGAGGAGTTTCAC	CATCAATTACATCATCCATCTGCATGC
*Mitf ex1mc*	CGACAAGCTTATGAACCGGCTTTTCCTG	
*Mitf ex1e*	TCACAGAGGTTAGTAGGTGGATGGG	
*Mitf ex1h*	GGCGCTTAGATTTGAGATGC	
*Mitf ex1m*	GAGGACTAAGTGGTCTGCGG	
*Mitf ex1b*	CCTGAGCTCACCATGTCCAAAC	
*Prss34*	GCTGATGAAAGTGGTCAAGATCATCCG	AGGAGTGAATGCATCAATATGAGTGGCTG
*Prss28*	GTACCGTGTTCATGGCCTCT	TGACTTTGGATGCAGTGAGC
*Prss29*	GTCAAGCTGCCCTCTGAGTC	TGGTTGCCTGCACATAACAT
*Tpsab1*	ATGACCACCTGATGACTGTGAGCCAG	AGGAACGGAGGTCATCCTGGATGTG
*Ube2i*	GAGGCTTGTTCAAGCTACGG	GTGATAGCTGGCCTCCAGTC
*Cacna1h*	CCTTTCTCAGCGTCTCCAAC	GCCACAATGATGTCAACCAG
*Polr2a*	CTGGACCCTCAAGCCCATACAT	CGTGGCTCATAGGCTGGTGAT

**Table A2:** Primer sequences for ChIP-qPCR analyses.

Primers		Sequences (5′ to 3′)	
		Forward	Reverse
***Tpsb2***	−72.8 kb	AACCTTCGACGTGACCTTTG	GGCACAGGATTTGTGAGACC
	−67.9 kb	TCAGTTGGCAGGTTTCTGTG	ACCAGTCAGGGCAAGTTCAC
	−63.4 kb	CTTACTGCTTTGGCCTGGAG	TATGAATTTGGAGGCGATCC
	−34.0 kb	AGCCTCCTAAGGGTCAGAGC	CCGCGATATTATCTGCACCT
	−1.1 kb	GGAGGTCACACTGCAGGATT	AATGGGTAACAGCGTCGTTC
	+2.6 kb	CTTGCCTTCCTTGTCCTCTG	AGAAGAGAGGGAGCCACACA
	−81.3 kb	GTGAGGCCCATTCAAAAGAA	CTAGGGAACCAGATGCCAAA
	−75.9 kb	GAGGACCCAAGTGAGGTTCA	AGAAGCCCCTAGGAGTGAGC
	−2.4 kb	CAGTCCAACTGCACCAACC	CACCCTCAGTTGCCTCCTCA
*Gata2*	−3.9 kb	GAGATGAGCTAATCCCGCTGTA	AAGGCTGTATTTTTCCAGGCC
	−27.7 kb	TGCCATGCCGGATATATTTTG	ACTAGCACGTGTGGCACAGTG
*Kit*	−114 kb	CGTGCACACAGGTTTGTTTC	TGCTGAGATGTGGCAATAGG

**Table A3:** Sequences of oligonucleotide used for CRISPR Cas9 genome editing.

Applied Oligonucleotide		Sequences of Oligonucleotide (5′ to 3′)
***sgRNAs***	5′-fwd	CACCGTCTTACTGTAGATTTAAGC
	5′-rev	AAACGCTTAAATCTACAGTAAGAC
	3′-fwd	CACCGACATTAGAACACTATTAGTA
	3′-rev	AAACTACTAATAGTGTTCTAATGTC
*5′ arm*	Fwd	TAATGGGCCCACAGGGCCTCATTTTGTAGG
	Rev	ACCGATCGATGCTTAAATCTACAGTAAGAC
*3′ arm*	Fwd	TATGGGATCCACTACTTTTACACTGCCTCA
	Rev	ACTTGCGGCCGCTACATGTTGGCACAAGCCTG
